# In this issue of *Current Oncology*

**Published:** 2007-08

**Authors:** M. McLean

We are delighted to inform the readership of *Current Oncology* that Dr. Richard J. Ablin, from the University of Arizona College of Medicine and the Arizona Cancer Center, has agreed to join Dr. Phil Gold as co-deputy editor. We welcome Richard, who has served on the editorial board of *Current Oncology* since 1998 and who is currently co-editor with Phil Gold of our section on *Updates and Developments in Oncology.*

In their respective roles, Phil and Richard will work together, with the help of our section editors, to expand the base of submissions from across Canada and the United States. In this regard, we look to you, our readership, for input about the kind of material that you would like to see—and indeed for original articles for publication in your specific areas of interest and expertise, and for reviews of areas that have experienced rapid progress on the clinical side.

Furthermore, Phil and Richard, together with other members of our staff, will be interfacing with industry and other stakeholders in the oncology community throughout North America while promoting *Current Oncology* and working to further enhance the impact value of the journal.

We anticipate that these exciting changes will bring *Current Oncology* to the next level, thereby filling a niche for not only for specialists in clinical oncology, but also for family physicians who have taken on major responsibilities in meeting the needs of cancer patients. In the process, we trust that *Current Oncology* will become an invaluable resource for the Canadian oncology community.

The gene for human epidermal growth factor 2 is amplified in approximately 20% of breast cancers, and the amplification is the primary mechanism for her2 protein overexpression. Overexpression of her2 is predictive of response to particular therapies, including trastuzumab (Herceptin: Genentech, San Francisco, California, U.S.A.) treatment in the meta-static and adjuvant settings, and updated Canadian guidelines for her2 testing are published in this issue of the journal. Two additional clinical practice guidelines are also included: one on the management of solitary brain metastasis, and another on the value of ifosfamide-based combination chemotherapy in advanced soft-tissue sarcoma. Stephen Sagar, in his role of section editor of *Integrative Therapies,* introduces a manuscript from Edzard Ernst titled “Homeopathy for cancer?” describing the evolution of the art of homeopathy over the last 200 years or so. And the National Cancer Institute of Canada (ncic) Clinical Trials Group’s article “Phase ii testing of sunitinib: the National Cancer Institute of Canada Clinical Trials Group IND Program Trials IND.182–185” leads off a new section of selected *Canadian Centre Activities.*

